# Levels of Ethylene Oxide Biomarker in an Exposed Residential Community

**DOI:** 10.3390/ijerph17228646

**Published:** 2020-11-21

**Authors:** Emily Szwiec, Lee Friedman, Susan Buchanan

**Affiliations:** Division of Environmental and Occupational Health Sciences, School of Public Health, University of Illinois at Chicago, Chicago, IL 60612, USA; szwiec1@uic.edu (E.S.); lfried1@uic.edu (L.F.)

**Keywords:** ethylene oxide, biomarkers, community exposure

## Abstract

The purpose of this study was to examine whether there is a difference in ethylene oxide (EtO) biomarker levels based on residential proximity to facilities emitting EtO, a carcinogen. We recruited residents living near two EtO-emitting facilities and administered a questionnaire on items such as address and length of residency, smoking habits, occupational exposures to EtO, and demographics. We also collected venous blood samples to measure an EtO biomarker, hemoglobin adduct N-2-hydroxyethyl-valine (HbEO), and cotinine, a metabolite of nicotine. Questionnaires and blood samples were collected from 93 participants. The overall geometric HbEO adduct level was 35.0 pmol/gmHb and for nonsmokers it was 29.7 pmol/gmHb. Mean HbEO adduct levels were not significantly associated with sex, age, race, BMI, or education level. HbEO adduct levels for nonsmoking participants who lived in a neighborhood approximately 0.8 km from one of the facilities were significantly higher compared to persons living farther away (*p* < 0.001). These results suggest that facilities that emit EtO may put nearby communities at risk of cancer and other associated health outcomes.

## 1. Introduction

Ethylene oxide (EtO) is a colorless, odorless gas used in industrial settings and as a sterilizer of medical equipment. Its most common use is as a precursor for other chemicals. Under the US Environmental Protection Agency’s Clean Air Act, EtO is one of 189 recognized hazardous air pollutants, and commercial sterilization and fumigation operations must adhere to standards limiting emissions.

Occupational-based studies have associated high-dose EtO exposure with acute health outcomes such as nausea, vomiting, bronchitis, pulmonary edema, emphysema, and miscarriage [1,2,3]. Long-term exposures at lower levels over periods of several months to years may cause irritation of the eyes, skin, and respiratory passages as well as headache, nausea, memory loss, and numbness [2,3]. The National Toxicology Program, USEPA, and the International Agency for Research on Cancer have all determined that EtO is carcinogenic [4,5,6]. Exposure to EtO may increase the risk of breast cancer, leukemia, and lymphoma [7,8].

In 2016, the USEPA lowered its inhalation unit risk value for EtO in recognition of EtO’s carcinogenicity at very low doses [9]. In August 2018, the USEPA released its most recent National Air Toxics Assessment (NATA) based on data from 2014 [9]. NATA is a screening tool that identifies which pollutants and emission sources may pose risks to public health, and is used to instigate further investigation by agencies. Many local permitting and emissions standards for EtO have not changed in response to the newly recognized cancer risk at lower doses. As a result, NATA’s analysis identified 18 different counties across 12 states where EtO air emissions were at levels that posed cancer risks higher than one in ten thousand people, considered an unacceptable risk according to the USEPA [10]. One of these identified counties is Lake County, Illinois, located north of Chicago, which is home to two facilities that emit EtO. One of the facilities sterilizes medical equipment, the other manufactures chemicals. The two EtO-emitting facilities are approximately 4.8 km from each other, and residents were recruited who lived in neighborhoods proximate to the facilities.

According to the USEPA’s latest Toxic Release Inventory(TRI) reports, the chemical production facility (Facility A) in Lake County released 1062 pounds (482 kg) of EtO in 2018—622 pounds (282 kg) from point sources and 440 pounds (200 kg) from fugitive sources [11]. There is no information available on emissions for 2019; however, the Illinois EPA drafted a permit for the facility—to take effect in 2020—limiting their EtO emissions to 110 pounds (50 kg) a year with no more than 60 pounds (27 kg) from fugitive sources [12]. The sterilization facility in Lake County (Facility B) does not meet the TRI reporting requirements, but a press report notes that the total emissions from the facility were 3058 pounds (1387 kg) in 2014 and 2863 pounds (1299 kg) in 2017 [13].

According to the USEPA’s Toxic Releases Inventory, there are no other known sources of EtO in Lake County, Illinois [11]. We conducted a pilot surveillance of exposure to ethylene oxide in a nonrandomized sample of residents living near the two EtO-emitting facilities. Volunteer participants completed a short exposure interview and we collected blood samples for an EtO biomarker, hemoglobin adduct N-2-hydroxyethyl-valine (HbEO) [14,15], and cotinine, a metabolite of nicotine [16,17].

## 2. Materials and Methods

The public health surveillance project was conducted in Lake County in August 2019. Recruitment was performed by distributing fliers in local neighborhoods and via social media posts, both facilitated by a local community activist group. The only eligibility criterion was that participants were required to have lived at their residence for the past four months, corresponding to the biologic half-life of the EtO biomarker in humans.

Data were collected during two sessions over one weekend at a local school. Participants were seen on a first-come, first-serve basis. They were administered a questionnaire that included items on smoking habits, including secondhand smoke exposure, marijuana use, and vaping; average hours spent in the home each day of the week; length of time at current residence; how often windows were open while at home; occupational exposures to chemicals; weight and height (to calculate body mass index (BMI)); and demographics, including race. Race and ethnicity categories in the study questionnaire were the same as those used in the US Census. The survey also included yes/no questions related to common symptoms seen with high-dose EtO exposure. The blood samples were tested for an EtO biomarker, HbEO (LOD = 12.9 pmol/gmHb) [14,15], and cotinine (LOD = 0.015 ng/mL), a metabolite of nicotine [16,17]. Measuring cotinine was important because smoking tobacco and exposure to secondhand smoke can result in HbEO adduct levels that are 5–10 times higher than levels in nonexposed persons [16,17]. Participants with cotinine levels <10 ng/mL were identified as nonsmokers [16,17]. Therefore, the association between residential distance from the facilities and adduct level was calculated for nonsmokers only. While smoking history was included in the survey, because of the high EtO levels in smokers, it was critical to definitively determine smoking status, so the cotinine level alone was used as a marker for smoking status in our analyses.

We developed an ordinary least squares regression model with covariates to analyze hemoglobin adducts of EtO (pmol/gHb) which was near-normally distributed. We ran a second sensitivity model using the log-transformed hemoglobin adducts of ethylene oxide (pmol/gHb) levels. We used SAS software for all statistical analyses (v.9.4; Cary, NC, USA).

## 3. Results

Questionnaires and blood samples were collected from 93 participants. Fifty-two participants (56%) were female and 40 (43%) were male (Table 1); one was unreported. The mean age was 53.9 years, and most participants (77.4%) identified as non-Hispanic white, 8.6% as Black/African American, 5.4% as multiracial, 4.3% as Asian, 3.2% as Hispanic/Latino, and 1% were unspecified. There were six children under the age of 15; the youngest was 7 years old. Over 40% were college graduates or higher. BMI was calculated using self-reported weight and height, and BMI categories were based on the Centers for Disease Control and Prevention standards. Eighty-five participants identified as nonsmokers. Of the eight smokers, six reported smoking every day (smokers identified by cotinine level are shown in Table 2). The mean number of years living at current address was 16.2 years, and the mean number of persons living in the home was 2.7 (data not shown). There were no statistically significant differences in adduct results within age categories (including for children under 15 years old), BMI categories, race/ethnicity, education level, or symptoms. The most frequently reported general health symptoms were nasal irritation, headaches, and fatigue/lethargy (data not shown). Upon reviewing the occupational exposures to chemicals, there were none identified that would result in higher than usual exposure to EtO, so these exposures were not included in the analysis.

**Figure 1:**
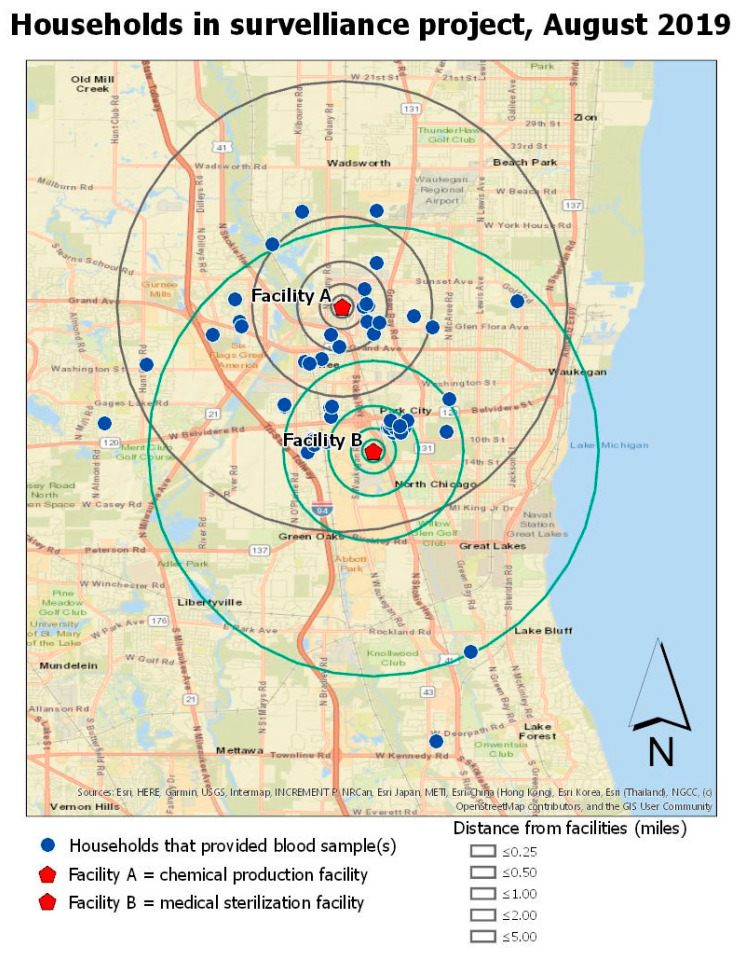
Map of households that participated in the surveillance project and their proximity to Facilities A and B.

Figure 1 shows the locations of participant households in relation to the two EtO-emitting facilities. A group of households clustered nearest to Facility B (approximately 0.8 km from the facility) was named Zone 1.

HbEO adduct results by exposure variable are shown in Table 2. The overall geometric mean HbEO adduct level was 35.0 pmol/gmHb (CI 95%: 30.3, 40.6); the median was 29.9 pmol/gmHb. Among nonsmokers, the geometric mean HbEO adduct level was 29.7 pmol/gHb (CI 95%: 27.1, 32.6) with a range of 13.3 to 295.0 pmol/gHb. For participants with cotinine levels ≥10 ng/mL (presumed smokers), the geometric mean HbEO adduct level was 163.5 pmol/gHb (CI 95%: 77.0, 347.5); range: 15.4 to 333.0 pmol/gHb. Adduct levels were significantly higher in smokers compared to nonsmokers (*p* < 0.001). For nonsmoking participants who lived in Zone 1, the geometric mean level of HbEO adducts was 37.2 pmol/gmHb (CI 95%: 26.9, 51.6). For nonsmoking participants who lived farther away from the facility, the geometric mean HbEO adduct level was 28.1 pmol/gmHb (CI 95%: 25.8, 30.5).

For both statistical models (ordinary least square regression and sensitivity model with log-transformed data), the adjusted analyses with individual covariates, as well as a priori knowledge, were used to determine inclusion of covariates in the final models. A two-sided *p*-value less than 0.05 was considered statistically significant. No evidence of multicollinearity among the final independent variables was indicated in any of the models based on evaluation of standard error(SE) and evaluation of variance of inflation and tolerance tests. Covariates included in the final model were education level, cotinine level, frequency of open windows at home, and reporting frequent activities adjacent to the sterilization facility (e.g., work or visiting family).

There was no evidence of an association between adduct levels and Facility A. Therefore, the rest of the analysis focused on residential distance from Facility B. The analysis of the association between distance of residence to Facility B and EtO adduct level revealed a cluster of homes that we named Zone. 1 The hemoglobin adduct levels from persons living in Zone 1 were on average 54.8 pmol/gmHb (SE= 12.1; *p* < 0.001) higher than persons living in other areas farther from the plant. In the model using the log-transformed data, persons living in Zone 1 continued to demonstrate significantly higher hemoglobin adducts of ethylene oxide (B = 0.63; SE = 0.13; *p* < 0.001). No other covariates were significantly associated with blood adduct levels or demonstrated modification or confounding effects.

## 4. Discussion

This surveillance project found that HbEO adduct levels were significantly higher among residents living closest to one of the two facilities that emit EtO compared to those living farther away. These results suggest that facilities that emit EtO may put nearby communities at risk of cancer, given that risk from exposure to carcinogens is considered a linear, non-threshold relationship—that is, there is no “safe level” of exposure to EtO. Facilities like the ones in Lake County, Illinois should either not be located near homes, schools, businesses, parks or other areas frequently used by the public, or local authorities should require the installation of ventilation capture systems that have been demonstrated to reduce air emissions to near-zero emission levels. 

The recently published National Health and Nutrition Examination Survey (NHANES) results from 2013 to 2014 and from 2015 to 2016 [18] found geometric mean HbEO adduct levels among nonsmokers of 27.8 pmol/gmHb and 27.0 pmol/gmHb, respectively (Table 2). Our results should be interpreted as surveillance data only since the participants were not randomly selected and the total number of participants was relatively small. Nevertheless, our result for nonsmokers living farther from the EtO-emitting facilities (28.1 pmol/gmHb) is comparable to the NHANES geometric mean, suggesting that those living farther from the facility are experiencing EtO exposure similar to the general US population. On the other hand, the geometric mean for nonsmoking participants living in Zone 1 in Lake County was 37.2 pmol/gmHb, which falls in the 75th to 90th percentile range of the most recent NHANES samples [18].

This small-scale surveillance of a convenience sample did not assess the correlation of local EtO air emission levels with residents’ adduct levels. Moreover, the sample size was small and not randomly selected in a blinded fashion, and therefore these results cannot be generalized to the entire exposed community. The monitoring of local air levels for EtO has been limited but is ongoing. In order to definitively determine the relationship between ambient air levels and residential exposure, a larger investigation should be conducted that would include more detail about time spent in the home and ambient air levels at various times of day and seasons of the year. Notably, due to its molecular weight, ethylene oxide is known to collect at ground level and has a half-life of 69 days during summer months and 149 days during winter months [19,20]. Therefore, ambient levels may be higher closer to the ground than at the level of the emission stacks. In addition, the use of wind-rose patterns is important when assessing community exposures to air contaminants. We did not obtain wind-rose patterns for the areas around the two facilities for this brief analysis, but we know based on local meteorological data that the prevailing wind direction is toward the northeast [21].

## 5. Conclusions

These results suggest that facilities that emit EtO may put nearby communities at risk of cancer and other associated health outcomes. The overall geometric mean HbEO adduct level was 35.0 pmol/gmHb, and for non-smokers it was 29.7 pmol/gmHb. HbEO adduct levels for nonsmoking participants who lived in a neighborhood approximately 0.8 km to the northeast of one of the facilities were significantly higher compared to persons living farther from the plant (*p* < 0.001). 

This surveillance project is the first to analyze ethylene oxide hemoglobin adducts of residents living near facilities emitting EtO, allowing a snapshot of nonoccupational exposures to EtO—an area which has not been well studied. As concern around EtO and communities’ exposure to this carcinogen grows, a research protocol should be developed for future investigations to definitively determine whether facilities that legally emit EtO increase cancer risk for residents living nearby.

## Figures and Tables

**Table 1:** Hemoglobin adduct N-2-hydroxyethyl-valine (HbEO adduct) results (in pmol/gmHb) by demographic variable.

	*N* (%)	Mean Ethylene Oxide Adducts—pmol/gHb (Standard Deviation)	*p*-Value *
**Sex**			
Female	52 (55.9%)	47.0 (sd = 60.4)	0.506
Male	40 (43.0%)	56.6 (sd = 77.0)	
Unspecified	1 (1.1%)	~	
**Mean Age in Years (sd)**	53.9 (sd = 19.2)		
0 to 14 yrs	6 (6.5%)	31.0 (sd = 7.5)	0.356
15 to 24 yrs	2 (2.2%)	40.1 (sd = 22.2)	
25 to 34 yrs	5 (5.4%)	74.7 (sd = 87.0)	
35 to 44 yrs	16 (17.2%)	77.4 (sd = 104.4)	
45 to 54 yrs	17 (18.3%)	41.3 (sd = 47.6)	
55 to 64 yrs	17 (18.3%)	73.1 (sd = 95.7)	
65 to 74 yrs	20 (21.5%)	31.2 (sd = 12.5)	
Unspecified	10 (10.8%)	~	
**Mean Body Mass Index**	27.7 (sd = 6.8)		
BMI less than 25	35 (37.6%)	38.1 (sd = 47.0)	0.115
BMI 25 to 29: overweight	33 (35.5%)	70.4 (sd = 89.3)	
BMI 30+: obese	25 (26.9%)	43.5 (sd = 54.0)	
**Race/Ethnicity**			
Asian	4 (4.3%)	43.5 (sd = 18.7)	0.341
Black/African American	8 (8.6%)	96.5 (sd = 115.0)	
Hispanic/Latino	3 (3.2%)	24.9 (sd = 5.3)	
Multiracial	5 (5.4%)	31.5 (sd = 7.5)	
Non-Hispanic white	72 (77.4%)	49.1 (sd = 65.5)	
Unspecified	1 (1.1%)	~	
**Highest Education Level**			
Grade 8 or below	2 (2.2%)	32.3 (sd = 1.6)	0.257
Some high school	2 (2.2%)	31.3 (sd = 25.4)	
High school graduate or equivalent	15 (16.1%)	77.4 (sd = 97.1)	
Some college	20 (21.5%)	58.4 (sd = 77.3)	
Technical or trade school	7 (7.5%)	97.0 (sd = 120.6)	
College graduate	21 (22.6%)	37.6 (sd = 45.2)	
Graduate school or higher	20 (21.5%)	31.5 (sd = 13.8)	
Unspecified	6 (6.5%)	30.9 (sd = 8.4)	

* *p*-value based on ANOVA test comparing mean hemoglobin adduct levels across categories.

**Table 2:** HbEO adduct results (in pmol/gmHb) by exposure and compared to US NHANES.

	Geometric Mean (CI 95%)	Median	Range	*p*-Value
Overall Study Participants (*n* = 93)	35.0 (30.3, 40.6)	29.9	13.3–333.0	
Smokers (*n* = 9 *)	163.5 (77.0, 347.5)	230	15.4–333.0	
Nonsmokers (*n* = 84)	29.7 (27.1, 32.6)	29.25	13.3–295.0	<0.001
Nonsmokers in Zone 1 (*n* = 17)	37.2 (26.9, 51.6)	31	19.5–295.0	
Nonsmokers outside of Zone 1 (*n* = 67)	28.1 (25.8, 30.5)	27.7	13.3–63.1	<0.001
NHANES 2013–2014 (geometric mean) [18]	27.8	28.3		
NHANES 2015–2016 (geometric mean) [18]	27.0	25.8		

* One participant identified as a nonsmoker but had elevated cotinine level so was considered a smoker. NHANES: National Health and Nutrition Examination Survey.
